# The application of mesoporous silica nanoparticles as a drug delivery vehicle in oral disease treatment

**DOI:** 10.3389/fcimb.2023.1124411

**Published:** 2023-02-14

**Authors:** Lixin Fang, Huoxiang Zhou, Long Cheng, Yiyi Wang, Fei Liu, Suping Wang

**Affiliations:** ^1^ Stomatology Center, The First Affiliated Hospital of Zhengzhou University, Zhengzhou, China; ^2^ The Academy of Medical Sciences, Zhengzhou University, Zhengzhou, China; ^3^ Laboratory of Microbiology and Immunology, Institute of Medical and Pharmaceutical Sciences & the Beijing Genomics Institution (BGI) College, Zhengzhou University, Zhengzhou, China; ^4^ Henan Key Laboratory of Child Brain Injury and Henan Pediatric Clinical Research Center, The Third Affiliated Hospital and Institute of Neuroscience, Zhengzhou University, Zhengzhou, China

**Keywords:** mesoporous silica nanoparticles (MSNs), drug delivery system, biofilm, dental caries, dentin hypersensitivity, oral squamous cell carcinoma, bone regeneration

## Abstract

Mesoporous silica nanoparticles (MSNs) hold promise as safer and more effective medication delivery vehicles for treating oral disorders. As the drug’s delivery system, MSNs adapt to effectively combine with a variety of medications to get over systemic toxicity and low solubility issues. MSNs, which operate as a common nanoplatform for the co-delivery of several compounds, increase therapy effectiveness and show promise in the fight against antibiotic resistance. MSNs offer a noninvasive and biocompatible platform for delivery that produces long-acting release by responding to minute stimuli in the cellular environmen. MSN-based drug delivery systems for the treatment of periodontitis, cancer, dentin hypersensitivity, and dental cavities have recently been developed as a result of recent unparalleled advancements. The applications of MSNs to be embellished by oral therapeutic agents in stomatology are discussed in this paper.

## 1 Introduction

**Figure 1:**
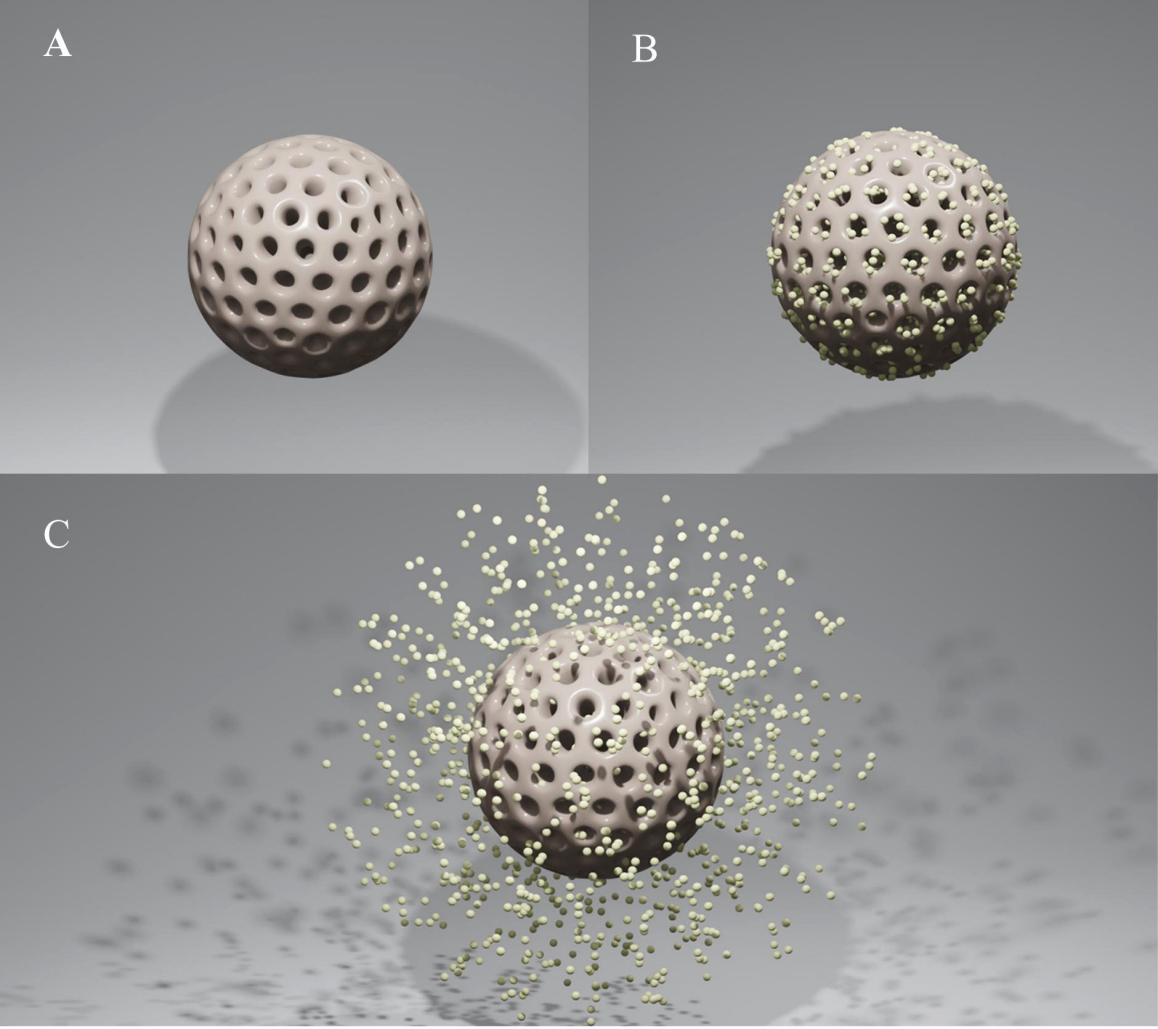
**(A)** Mesoporous silica nanoparticle (MSN). **(B)** MSN as a vehicle for drugs. **(C)** The agent’s release of drug-loaded MSNs.

Mesoporous silica nanoparticles (MSNs) have drawn a lot of interest as novel therapeutic nanocarriers because of their ability to release a variety of drugs at the desired place in response to external stimuli. MSNs are more advantageous choices for drug loading as compared to other nanocarriers because of their tunable morphologies, mesostructures, and porosities, as well as their superior biocompatibility and simplicity of functionalization. Furthermore, mesoporous materials' high surface areas and large pore volumes enable them to hold more medications or molecules (**Figures 1A, B**). Thanks to their advantage in functionalization, they also provide new opportunities for medicinal synthesis in combination with other drugs. As a result, drug transfection into specific areas dramatically reduces side effects, and higher drug loading directly enhances therapeutic benefit. MSNs, one of the most promising nanocarriers, also have other exceptional benefits, including easy and affordable production, stability, dissolvability, biocompatibility, and biodegradability. With all of these attributes, MSNs give medications the needed solubility and stability in solution. This delivery system responds to particular micro-circumstances (such as pH, temperature, light, and magnetic and electric fields), which are the foundation of innovative treatment approaches in stomatology (**Figure 1C**). Targeted delivery also exhibits an effective and safe therapeutic strategy by greatly increasing the drug concentration in the treatment region and reducing the negative effects on adjacent normal tissue. Highly cytocompatible MSNs make it possible to load drugs in the following fields: 1) Combination with different drugs to improve their poor performance and allow responsive drug delivery simultaneously. 2) Provide the shared nanoplatform of drugs to control biofilm and cure infectious diseases synergistically while avoiding drug resistance. 3) Provide multiple medications with a shared nanoplatform to address conditions such as tumors and dental hypersensitivity. 4) MSNs with excellent surface properties and porosity have proven to be attractive bioactive materials for bone regeneration. Targeted delivery and biocompatibility extend the utilizing scope of MSNs. The recent research developments on MSNs and the biological uses of MSNs in stomatology, including antibiofilm, antitumor, reducing dentin sensitivity, and stimulating osteogenesis for bone regeneration, are summarized here.

## 2 Mesoporous silica nanoparticles as the vehicle for oral drugs

### 2.1 Mesoporous silica nanoparticle as a vehicle for chlorhexidine

As well as Gram-positive and Gram-negative organisms, chlorhexidine (CHX) is effective against a variety of fungi, facultative anaerobes, and aerobes. CHX adheres to the microorganism’s cell wall and causes a leaking of internal components, which is how it works (Fardal and Turnbull, 1986). CHX is regarded as the “gold standard” to assess the antibacterial effect due to its broad-spectrum antibacterial activity, and its application has been thoroughly investigated, for instance, in mouthwash, dentin adhesives, and repair supplies. Previous *in vitro* and *in vivo* research has shown that MSNs have a promising capacity as a drug delivery vehicle for antibacterial agents (Hetrick et al., 2009; Slomberg et al., 2013). In order to enhance the antibiofilm efficiency and lengthen the antibacterial duration, CHX was encapsulated into MSNs. CHX@MSNs were able to penetrate the *Streptococcus mutans* biofilm matrix and closely interact with microbes to improve the antibiofilm efficiency (Li et al., 2016). CHX released from CHX@MSNs inhibited biofilms even after 50 h. Additionally, the bacterial resistance to CHX was overcome by adding additional drugs to MSNs to produce synergistic antibacterial effects (Lu et al., 2017; Lu et al., 2018). Moreover, CHX@MSNs were modified into dentin adhesives to reduce cariogenic bacteria and impede biofilm permeation because a cariogenic bacteria-created acidic environment might cause pH-sensitive CHX@MSNs to release CHX (De Munck et al., 2009; Akram et al., 2021). According to recent findings, MSNs had great potential in dental restorative materials. CHX@MSNs could carry significant CHX when added to glass ionomer cement or composites, which boosted their antibacterial and antibiofilm activities without sacrificing their mechanical performance (Zhang et al., 2014; Yan et al., 2017). In contrast, directly mixing CHX into composites resulted in a burst release over a short period of time and produced a porous surface that encouraged bacterial adhesion and biofilm formation. Furthermore, MSNs can also serve as a co-delivery platform for simultaneously loading different agents to combat drug resistance. CHX-loaded silver (Ag)-decorated MSNs (Ag-MSNs@CHX) have been shown to exert more effective antibiofilm effects and remarkably reduce the toxicity of CHX in oral epithelial cells (Lu et al., 2017; Lu et al., 2018).

### 2.2 Mesoporous silica nanoparticle as a vehicle for silver nanoparticles

Ag nanoparticles (AgNPs) are frequently employed in stomatology because they can enter cells through their membranes and cause cell lysis. The electrostatic adsorption between the bacterial cell wall and AgNPs might kill bacteria by preventing the synthesis of proteins and deactivating respiratory enzymes (Santos-Beneit, 2015; Khubchandani et al., 2022). For a wider variety of applications, AgNPs were added to denture materials or orthodontic adhesives (Monteiro et al., 2009). For instance, Ag had been directly mixed into polymethylmethacrylate (PMMA); however, the addition did not confer materials with better mechanical properties and long-lasting antibacterial effects (Mohamed Hamouda, 2012). MSNs protected AgNPs from aggregation, and the controlled drug release lessened the cytotoxicity of AgNPs (Lok et al., 2007; Chen et al., 2016; Jin et al., 2018; Liu et al., 2018). Therefore, they were employed as nanocarriers to load AgNPs (Ag-MSNs) and then incorporated with PMMA, which showed sustained flexural strength and microbial anti-adhesive effects for 14 days (Jo et al., 2017). AgNPs had also worked with remineralization agents to stop dental caries (Carrouel et al., 2020). MSNs coated with bioactive glasses (BAGs) induced dentinal tubule occlusion and remineralization, and the addition of AgNPs would give BAG-coated MSNs antibacterial capabilities. Ag-MSN-based nanomaterials showed considerable promise in the treatment of dentin hypersensitivity and caries prevention, since Ag-BAG@MSN efficiently blocked the dentinal tubule following the acid challenge and inhibited the growth of bacteria (Tian et al., 2014; Jung et al., 2019). MSNs provide Ag with great stability, sustained antibacterial efficacy, and significant safety. The antimicrobial effectiveness of AgNPs is increased, and the utilization range is expanded after being loaded into MSNs.

### 2.3 Mesoporous silica nanoparticle as a vehicle for quaternary ammonium salts

Through electrostatic interaction, quaternary ammonium salts (QASs) cling to the negatively charged bacterial cell membrane and kill bacteria in contact by integrating a hydrophobic alkyl tail into the lipid bilayer of the membrane (Zhang et al., 2018). They are frequently used in dental materials including dental adhesives, pit and fissure sealants, and dental implants. However, the contact-killing mechanism will not work if bacteria do not come into direct contact with materials, and the protein linked to the surface of the material will further reduce the killing effectiveness. A way to enhance the antibacterial activity is to exploit the synergistic effect by putting dual drugs on the same nanovehicle. MSNs are frequently employed in drug-loading fields because of their excellent drug-loading capacity and biocompatibility as well as their easily functionalized surface. MSNs decorated by Ag and QAS (Ag/QAS-MSNs) were created to combat head and neck cancers (HNCAs) and follow-up infections simultaneously in light of the antibiofilm and anticancer capability of Ag and QASs (Ito et al., 2009; Meena et al., 2017; Tang and Zheng, 2018; Deshmukh et al., 2019; Ahn and Park, 2020; Eid et al., 2020; Zhang et al., 2022). The findings showed that Ag/QAS-MSNs prevented the formation of bacterial colonies for at least 14 h mostly as a result of the sustained release of Ag+ and QAS from Ag/QAS-MSNs, which directly caused membrane damage and cell death. Comparing Ag/QAS-MSNs to QAS-MSNs, bare AgNPs, and pure QAS, Ag/QAS-MSNs also demonstrated the greatest antibacterial activity in a concentration-dependent manner (Zhang et al., 2022). AgNPs@MSNs treated with quaternary ammonium polyethyleneimine (QPEI), one of the QASs, were able to overcome the electrostatic repulsion between AgNPs and bacteria. Results revealed that compared to Ag@MSNs and QPEI alone, Ag@MSN-QPEI had greater antibacterial activity and a longer bactericidal duration (Niu et al., 2021; Zhou et al., 2021). MSNs have a potential future in the treatment of HNCA with higher antibacterial activity against follow-up infections as vehicles for QASs to co-deliver other drugs.

### 2.4 Mesoporous silica nanoparticle as a vehicle for curcumin

Curcumin has outstanding antibiofilm, anti-inflammatory, and antitumor activities. It prevents *S. mutans* from adhering to extracellular matrices and tooth surfaces, perturbing membrane integrity and inducing entocyte leakage of *Streptococcus* (Tyagi et al., 2015; Pamukcu et al., 2022). Curcumin was safe enough, and oral treatment did not cause reproductive toxicity in humans even at 500 mg twice daily for 30 days (Soleimani et al., 2018). However, the insolubility in water, limited bioavailability, and instability in the biological environment of curcumin hampered its therapeutic use. Recently, studies had focused on curcumin-loaded MSNs (Cur-MSNs) due to their abilities to overcome the mentioned constraints of curcumin with a high encapsulation efficiency, protecting curcumin from premature leakage and providing a controlled drug release (Ribeiro et al., 2022). Numerous studies had been conducted to demonstrate both their effect on the infections and their use in cancer therapy. Due to the MSNs’ capacity to penetrate the matured biofilm matrix, curcumin can destroy the developed biofilms and suppress the development of biofilms with lower required doses and higher cytocompatibility after being repurposed by MSNs (Pamukcu et al., 2022). As the shared nanocarrier, MSNs could combine several antimicrobial components to increase antimicrobial impact. The compounds that AgNPs decorated and curcumin-charged MSNs were characterized by low hemolytic action and persistent growth-inhibiting impact on *Staphylococcus aureus* and *Escherichia coli* (Song et al., 2020). Furthermore, curcumin reduced the proliferation of tumor cells *via* targeting molecules expressed by cancer-relevant genes and increasing the production of intracellular reactive oxygen species (ROS) (Shanmugam et al., 2015; Hafez Ghoran et al., 2022). MSNs easily enter cells through phagocytosis, and the numerous silanol groups on their surface enable the controllable curcumin release, showing greater potential in reducing tumor cell proliferation (Liu et al., 2018; Zhou et al., 2018). Compared with free curcumin, Cur-MSNs showed higher cytotoxicity in HNCA cells (Sharifi et al., 2022). Additionally, targeted distribution by hyaluronic acid (HA)-modified or -aminated MSNs had improved anticancer efficacy in breast cancer cells and colon cancer cells (Ghosh et al., 2021; Liu et al., 2022). These studies demonstrated that curcumin had a stronger effect on bacteria and tumor after loading into MSNs.

## 3 The prospective application of mesoporous silica nanoparticles in stomatology

### 3.1 Dental caries

*S. mutans* is the predominant etiological pathogen that firmly adheres to tooth surfaces and plays a critical role in generating an acidic environment. This environment ensures the development of biofilms, demineralization of the teeth, and the onset of dental caries. CHX@MSNs that were synthesized by loading CHX in functionalized MSNs showed a long-term and stimuli-responsive release of agents, meaning that the lactic acid produced by *S. mutans* might burst the release of CHX from MSNs (Zhang et al., 2014; Lu et al., 2018). Secondary caries is the primary cause of dental composite repair failure. Dental composites releasing antibacterial agents effectively reduced secondary caries and inhibited cariogenic biofilms, which could extend the service life of composite restorations. Therefore, incorporating CHX@MSNs into experimental resin-based dentin adhesives and dental composite showed potent inhibition of planktonic growth and biofilm formation with excellent bonding strength and least nanoleakage. Compared with directly mixing CHX into composites, composites containing CHX@MSNs largely kept their mechanical properties and smooth surfaces, resulting in the accumulation of very few planktonic bacteria with deformed membranes on the surface of composite resin (Zhang et al., 2014; Akram et al., 2021). Adding zinc (Zn) to dental resin composites has attracted more and more attention, since it has no adverse effects on the esthetic performance of the resins. However, the release of Zn from zinc oxide (ZnO) might lead to the destruction of the ZnO fillers and impair the composition’s mechanical properties, and the difficulty in releasing Zn sustainably may have an impact on the composition’s long-term antibacterial performance (Wang et al., 2019). The prepared Zn-MSNs effectively address the mentioned problems with improved the mechanical and antibacterial properties of the dental resin composites (Alvarez et al., 2021). In addition, the presence of Zn-MSNs has no detrimental effect on the conversion, shrinkage, curing depth, and biocompatibility of dental resins, indicating the potential of MSNs in dental compositions that transport agents (Bai et al., 2020).

### 3.2 Dentin hypersensitivity

Dentin hypersensitivity is characterized by rapid acute pain in response to thermal, chemical, and physical stimulation. Dentin exposure from abrasion, acid erosion, and gingival recession results in dentin hypersensitivity. According to the most widely recognized theory—hydrodynamic theory—obturating the exposed dentinal tubules with biomaterials to lessen the flux will be effective in treating dentin hypersensitivity.

Acid resistance is necessary for biomaterials to maintain their stability over time in the face of everyday acid erosion, which may be impacted by the depth of ions deposited in tubules. The biomedical fields have extensively used small and well-dispersed MSNs that were packed with remineralization agents and deeply infiltrated into dentinal tubules without compromising dentin bond strength (Zhang et al., 2018). Nano-hydroxyapatite (nHAp) acted as the Ca^2+^ and PO_4_^3−^ reservoir that can facilitate crystal deposition and formation in demineralized portions of teeth. The dentinal tubules were blocked by the nHAp@MSN, which also prevented nHAp from dissolving without impairing the microtensile bond strength (MTBS) (Yu et al., 2016). However, applying remineralization agents alone would not be sufficient to manage the dentin surface, since exposed dentin was more prone to dental cavities. A natural extract derived from green tea called epigallocatechin-3-gallate (EGCG), which has versatile uses as an antibiofilm and anti-inflammatory agent, could be encapsulated into nHAp@MSNs to prevent caries by eradicating *S. mutans* biofilm. EGCG@nHAp@MSN was a multifunctional biomaterial for dentin hypersensitivity and caries by occluding dentinal tubules, reducing biofilm formation, and maintaining favorable acid-resistant stability (Yu et al., 2017). Bioactive glass nanoparticles (BGNs) relieved the discomfort of dentin hypersensitivity by occluding dentinal tubules and soft tissue regeneration (Jung et al., 2019). It was confirmed that the synthetic biocomposite material Ag-BGNs@MSN, which has a greater surface area, successfully induces remineralization, exerts antibacterial capability, and is a useful substance for the treatment of dentin hypersensitivity (Jung et al., 2019). Since tooth flaws frequently accompanied dentin discomfort, resin-based repair was required. Ag-BGNs@MSNs did not inhibit MTBS in *in vitro* research, but additional *in vivo* investigations are still needed to determine whether or not the material’s characteristics alter.

### 3.3 Periodontitis

Dental plaque plays a role in the etiology of periodontitis, which finally results in tooth loss by destroying the tissues supporting the teeth. However, due to the intricate tooth anatomy, mechanical debridement by scaling and root planing (SRP) to remove the subgingival plaque does not entirely eradicate germs, especially in deep pockets (Warinner et al., 2014; Zupancic et al., 2019). The unique therapeutic approach for periodontitis was made possible by MSNs, which enhanced medication concentration in the targeted tissue for the stimuli-responsive release and made it easier to kill bacteria in periodontal pockets (Hayes et al., 2018; Lin et al., 2020). In earlier research, MSNs were mechanically applied to prevent infections while being loaded with various chemicals, including CHX and antibiotics to eliminate biofilms (RR et al., 2019; AL et al., 2021). The host inflammatory response elicited by the subgingival dental biofilm also needs to be treated for the resultant irreversible destruction of the periodontium. Resveratrol (RSV; 3,5,4’-trihydroxy-trans-stilbene) has strong anti-inflammatory and antimicrobial effects, but its application is severely constrained by the poor water solubility, rapid decomposition, and short serum half-life (Bhattarai et al., 2016). The RSV-grafted MSN drug carrier could successfully extend its bioavailability in the local periodontal region, resulting in sustained pharmacological activity and removing RSV’s inherent cytotoxicity (Tan et al., 2022). For RSV’s anti-inflammatory effects and the ability to modulate glucose metabolism, MSN-RSV may also be able to alleviate diabetic periodontitis (DP) (El-Makaky and Shalaby, 2020). Diabetes mellitus (DM) impairs bone repair by increasing ROS production, which speeds up periodontal bone loss and makes bone regeneration in DP difficult (Hajishengallis et al., 2012; Mysak et al., 2014; Wang et al., 2023). Recent research used MSN-incorporated poly (D, L-lactide)-block-poly (ethylene glycol)-block-poly (D, L-lactide) (PPP) to achieve stepwise cargo release and emulate the cascade for diabetic periodontal bone regeneration, which can scavenge the overproduced ROS, regulate the diabetic microenvironment, and facilitate osteogenesis (Wang et al., 2023). In conclusion, MSNs offer flexible treatment plans for periodontitis that include elimination of the pathogens, reduction of inflammatory effects, and facilitation of osteogenesis.

### 3.4 Endodontic treatment failure

Endodontic treatment failure can be caused by a variety of factors, including the persistence of microorganisms, improperly cleaned root canals, and untreated canals (missing canals) (Alghamdi and Shakir, 2020). *Enterococcus faecalis* is the most common isolate from endodontic infections and is strongly linked to failed endodontic treatments because of its capacity to survive in extremely challenging conditions with limited nutrient availability and a high alkaline pH that can reach 11.5 (Stuart et al., 2006; Ozbek et al., 2009). Additionally, the mono-infection of *E. faecalis* in treated canals without synergistic assistance from other bacteria results in significant resistance to antimicrobial treatments. Incomplete removal of *E. faecalis* from the root canal by sodium hypochlorite (NaClO) and CHX highlights the need for more sophisticated strategies for thorough disinfection in endodontic treatments (Vianna et al., 2004; Eddy et al., 2005; Estrela et al., 2008). Sonodynamic therapy (SDT) relies on ultrasound (US) to activate the sonosensitizers and generate the ROS to obliterate bacterial infection (Serpe and Giuntini, 2015; Xu et al., 2017). MSNs are synthesized as the platform for conjugation with sonosensitizer protoporphyrin IX (PpIX) (MSNs@P) and Fe ions (MSNs@P-Fe) to initiate a Fenton action in order to destroy bacteria without having to worry about resistance (Pang et al., 2019; Guo et al., 2021; Wang et al., 2021). Compared with the commonly used NaClO irrigant, this new strategy (MSNs@P-Fe + 0.01% H_2_O_2_ + US) is highly efficient in eliminating *E. faecalis* infection by exploiting low-concentration H_2_O_2_ to generate highly toxic ROS without inducing notable cell toxicity. This technique with excellent tissue penetrability is noninvasive and site-confined, showing the MSN platform’s potential in the elimination of deeply ingrained infection. In addition, MSNs are selected as scaffolds in combination with hydrogel for the proliferation of human dental pulp stem cells (HDPSCs), and this new biopolymer scaffold improves the immigration and regeneration of HDPSCs to repair pulpitis (Wang et al., 2022).

### 3.5 Maxillofacial space infection

Fascial space infections are the common sequelae of odontogenic infections including periapical infection and pericoronitis (Singh et al., 2021). Patients with superficial dental infections typically experience localized pain and cellulitis, while those with deep infections or abscesses may experience swallowing and breathing issues. *S. aureus* is the dominating pathogenic bacterium of mouth floor cellulitis, a multispace infection that affects the sublingual, submental, and submandibular spaces with potentially life-threatening effects (Ogle, 2017). Selenium (Se) nanoparticles (SeNPs) are considered to be healthier and less toxic to healthy cells and have antibacterial effects. Incorporating Se into MSNs exhibits better antibacterial activity against *S. aureus*, and dispersibility is improved by preventing SeNP agglomeration (Chen et al., 2020). Methicillin-resistant *Staphylococcus aureus* (MRSA) biofilms pose a unique challenge in space infections due to the tolerance to various antibiotics. Proteins and environmental DNA (eDNA) make up the majority of the MRSA biofilm matrix, which makes it difficult for antibiotics to reach the deepest parts of the biofilm and precisely target cells (McCarthy et al., 2015). Meanwhile, immunosuppression increased the incidence of MRSA infection in patients with head and neck squamous cell carcinoma (HNSCC) following chemotherapy. Antibiotics delivered by nanoparticle-based carriers penetrate the biofilm better. In order to eliminate MRSA biofilms and target *S. aureus*, enzyme-functionalized MSNs are created. Results of cell viability and crystal violet staining demonstrate that the enzyme’s efficiency against *S. mutans* was further enhanced after immobilizing into MSNs (Devlin et al., 2021). Sortase A (SrtA), a membrane-bound cysteine transpeptidase, binds virulence-associated proteins to the bacterial cell wall (Nitulescu et al., 2017). Naturally derived compounds with poor water solubility are classified as sortase A inhibitors (SrtAIs), including quercetin (QC) and berberine chloride (BR). With the help of MSNs, SrtAI’s solubility can be increased, opening up new therapy options for superbugs with less hazardous side effects (Alharthi et al., 2022). When drugs are combined with MSNs, the antibacterial effect may be enhanced, the drug’s release time may be prolonged, the inherent cytotoxicity may be eliminated, and bacterial resistance may be addressed with great physiochemical performance.

### 3.6 Oral squamous cell carcinoma

Oral squamous cell carcinoma (OSCC) has great probability of metastasis that may lead to poor prognosis or even death. The main medications used in OSCC treatment include paclitaxel (PTX), 5-fluorouracil (5-FU), methotrexate, and cisplatin (Molin and Fayette, 2011). With a lower pH and higher temperature than normal tissue, the tumor has different properties. MSNs are the prospective carriers to treat malignancies because of their perceptive response to pH and temperature (Lu et al., 2010; Meng et al., 2010). It is possible to destroy tumor cells by inhibiting the supply of glucose and causing a redox reaction because tumor cells have higher glucose requirements and endogenous reducing agents than normal cells (Chen et al., 2020). The combination of starving therapy with MSNs is prospective to increase the effectiveness in treating tumors. Glucose oxidase (GOx) and PTX can interrupt the intracellular energy supply and elevate the endogenous H_2_O_2_ level of tumor cells, exhibiting an amplified effect. Therefore, GOx and PTX were co-delivered *via* the MSNs as a nanoplatform to induce better therapeutic effects against cancer (Du et al., 2019). In addition, 5-FU, which is frequently used to treat OSCC, has hematologic and digestive side effects, including anemia, thrombocytopenia, and leukopenia (Bui et al., 2020). This nanoplatform of MSNs could preferentially accumulate 5-FU in tumors to suppress tumor growth and avoid side effects (Lee et al., 2010; Lu et al., 2010). The outer membrane vesicle (OMV)-MSN-5-FU overcomes the mentioned drawbacks by reducing the cumulative drug release and prolongs the targeted action time to inhibit tumor proliferation and metastasis (Huang et al., 2022). Consequently, MSNs can circumvent the challenges associated with administering anticancer medications by delivering them to specific tissues to improve biocompatibility.

### 3.7 Bone regeneration in the oromaxillofacial region

Periodontitis, maxillofacial infections, and tumors cause varying degrees of bone abnormalities. The main treatments for jaw abnormalities mainly include autologous bone graft, allogeneic bone graft, and artificial substitute implantation. Opening up the second surgical area is invasive, although clinical vascularized autologous bone graft is mature in repairing maxillofacial defects. Artificial replacements with a microporous structure, a particular hardness, and the ability to induce cell differentiation can be used to heal bone abnormalities. MSNs conveying biological cues in a targeted and regulated manner can improve the behavior of osteoclasts and the mechanical qualities of the biomaterial by attaching MSNs to the titanium substrate’s surface (Rosenholm et al., 2016). MSNs are modified with a bone-forming peptide (BFP) to provide a slow-release mechanism for delivering osteogenic factors. Experiments demonstrate that BFP-laden MSNs (p-MSNs) with a sustained peptide release rate and better bioactivity could promote the osteogenic differentiation of mesenchymal stem cells (MSCs) and the spread of human osteoblast-like MG-63 for bone repair and regeneration (Luo et al., 2015). The MSNs were used to transmit genes and promote osteogenic differentiation. Bone morphogenetic protein-2 (BMP-2) plasmid DNA (pDNA) was combined with aminated MSNs (MSN–NH_2_), and the BMP-2 protein was produced by transfected MSCs, which demonstrated the potential of MSNs as a gene delivery system in bone regeneration (Kim et al., 2013). The *in vitro* cell cytotoxicity tests indicated that BMP-2 peptide-functionalized MSNs (MSNs-pep) is highly cytocompatible, and the osteoblast differentiation and bone regeneration of MSCs could be further enhanced after dexamethasone (DEX) was incorporated (Zhou et al., 2015). These systems also offer a nanoplatform on which to load various medications for efficient osteoblast development.

## 4 Conclusions and perspectives

MSNs offer interesting characteristics that can be used in combination with one another to enhance stomatology drug delivery. They also have significant potential for antibiofilm, tumor therapy, and combined therapy. Recent studies demonstrate that MSNs can improve the dissolution rate and bioavailability of the water-insoluble drugs by entrapping them in the mesopores and dispersing them with a large surface area. Moreover, MSNs functioning as nanoplatforms improve the antimicrobial effectiveness through combining various antimicrobial components. This co-delivery nanoplatform with several stimuli-responsive confers final compounds the abilities of antibacteria, antitumor, and bone regeneration of maxillofacial defects. The drug released from MSNs to targeted locations lowers the dosage with a longer half-life and improves the therapeutic effect (Esfahani et al., 2022). To help them evolve further, some important difficulties, such as the potential cytotoxicity and MSN excretion, must be resolved. Firstly, by raising the quantity of ROS, MSNs cause oxidative stress and apoptosis. Secondly, the therapeutic action will be limited by the residuals in the MSNs because only a portion of the medications will be released. Thirdly, due to the striking differences in the multistep MSN synthesis process, scaling up synthesis will face significant difficulties. The long-term therapeutic effect of MSN-based systems *in vivo* should be rigorously and extensively proven before the clinical translation of MSNs. Given the satisfactory resolution of these issues, MSN-based formulations may achieve exciting breakthroughs in the treatment of a variety of significant diseases and disorders.

## Author contributions

All authors contributed to generate the ideas presented in this review. LF wrote the original draft and all the co-authors reviewed and complemented the text. All authors contributed to the article and approved the submitted version.
